# Barriers and facilitators to implementing e-learning for infection management in primary care: a scoping review

**DOI:** 10.1186/s12875-026-03385-4

**Published:** 2026-05-19

**Authors:** Louise Bidstrup Jørgensen, Jette Nygaard Jensen, Caroline Skovsbo Clausen, Marius Brostrøm Kousgaard, Sif Helene Arnold

**Affiliations:** 1 Department of Public Health, The Centre of General Practice, University of Copenhagen, Copenhagen, Denmark; 2 Department of Clinical Microbiology, Copenhagen University Hospital - Herlev and Gentofte, Copenhagen, Denmark

**Keywords:** Primary care, Infection management, Implementation, E-learning, Scoping review

## Abstract

**Background:**

Infections are among the most common reasons for consultations in primary care, and appropriate infection management is essential to patient safety and antimicrobial stewardship. Educational interventions can improve diagnostic and prescribing practices, but in-person training is resource-intensive and difficult to scale. E-learning offers a flexible alternative; however, little is known about factors that influence its implementation in primary care.

**Objective:**

To identify barriers and facilitators to implementing e-learning interventions for infection management in primary care settings.

**Methods:**

A scoping review was conducted in accordance with established methodological guidance. Systematic searches across five databases was performed and updated in October 2025 to identify studies describing asynchronous, interactive e-learning interventions targeting infection management in primary care. Eligible studies were screened, and implementation determinants were extracted and mapped using the Consolidated Framework for Implementation Research (CFIR).

**Results:**

Seven studies published between 2017 and 2025 were included, with implementation determinants typically reported alongside feasibility or contextual findings rather than as primary outcomes. Most studies employed quantitative designs, and one used mixed methods. Reported determinants were mainly mapped to the CFIR Innovation and Individuals domains, highlighting the importance of intervention design, adaptability, and perceived relevance for professional roles. Barriers frequently related to misalignment between intervention content and participants’ level of expertise, platform complexity, and challenges related to IT infrastructure. Determinants related to organizational context, implementation processes, and external conditions were reported less frequently.

**Conclusions:**

The available evidence on the implementation of e-learning for infection management in primary care is limited and heterogeneous. Future research should include more robust implementation studies, including qualitative approaches, to better capture the important determinants for implementation at the individual, organizational, and system levels. Such insights are needed to inform the development and implementation of scalable and sustainable e-learning interventions in primary care.

## Introduction

Infections are among the most common reasons for consultations in primary care, and appropriate assessment and management are essential for ensuring safe and effective patient care [1, 2]. However, diagnostic uncertainty and inadequate diagnostic practices, including misconceptions regarding symptom evaluation, can influence clinical decision-making and lead to unnecessary or inappropriate treatment [3–7]. Such challenges have been associated with variation in diagnostic decisions, as well as difficulties in applying appropriate clinical criteria in everyday practice [3–7]. This may contribute to adverse patient outcomes and, especially when antibiotics are involved, contribute to increased antimicrobial resistance [5, 6, 8, 9]. Globally, antimicrobial resistance continues to pose a major public health threat, and improving diagnostic accuracy and treatment decisions in primary care settings is therefore increasingly important [10, 11]. In this review, infection management is understood as a broad set of clinical and organizational practices in primary care aimed at ensuring appropriate management of infectious conditions, including but not limited to practices related to antimicrobial stewardship and antimicrobial resistance.

Effective infection management includes strengthening knowledge, decision-making, and communication among healthcare professionals (HCPs) involved in day-to-day assessment and treatment, such as general practitioners (GPs), nurses, and other healthcare staff. Educational interventions have shown potential to improve infection management [12, 13]. However, in-person educational training in primary care presents challenges to scalability and sustainability. Thus, in-person training requires the simultaneous presence of both educators and HCPs from busy primary care settings, making it resource-intensive with respect to time, coordination, logistics, and staffing. The challenges are accentuated by high turnover rates for staff in many primary care settings, which creates a need for ongoing educational efforts to ensure sustainable change [14, 15]. Digital learning, particularly asynchronous and easily replicable e-learning, is increasingly recognized as a potential solution to overcome the limitations of in-person educational interventions [16–18]. This approach offers flexible access to training and scalability, making it well-suited for HCPs working in settings where attendance in traditional in-person education is challenging [17].

Despite this growing use of e-learning in healthcare, relatively little is known about the specific factors that influence its implementation in primary care. While previous studies suggest that e-learning has the potential to improve knowledge and professional skills [19, 20], these effects depend on successful uptake and sustained use in routine clinical practice. However, existing studies have focused on educational outcomes, while less attention has been given to the organizational and behavioral determinants that shape uptake and sustained use in everyday clinical setting [20]. Understanding these determinants is essential to ensure that e-learning interventions are designed and implemented in ways that support better antimicrobial stewardship.

On this background, we conducted a scoping review to identify barriers and facilitators for implementing e-learning on infection management in primary care settings. This scoping review methodology allows for systematic mapping of the breadth of existing evidence, including how determinants have been described across different healthcare contexts, and where knowledge gaps remain.

## Methods

This scoping review was conducted in accordance with an a priori protocol available at Open Science Framework [21]. The review followed the methodological guidance in the Joanna Briggs Institute (JBI) Manual for Evidence Synthesis [22]. Reporting adhered to the Preferred Reporting Items for Systematic Reviews and Meta-Analyses extension for scoping reviews (PRISMA-ScR) [23]. A completed PRISMA-ScR checklist is provided as Additional File 1.

### Eligibility criteria

Eligibility criteria were defined using the Population-Concept-Context (PCC) framework recommended by the JBI Manual for Evidence Synthesis [24].

#### Population

We included studies involving HCPs delivering patient care within primary healthcare settings. Studies involving students or non-patient-facing personnel were excluded.

#### Concept

Eligible studies described asynchronous, interactive e-learning interventions targeting infection management in adults (≥ 18 years), delivered as part of continuing medical education (CME) or other professional development activities.

For the purpose of this review, infection management was defined as the assessment, diagnosis, treatment, prevention, or stewardship of infectious conditions. During screening, studies were considered relevant if the e-learning intervention addressed one or more of these aspects.

Interventions combining more than two educational components (of which e-learning had to be one) or lacking an interactive e-learning element were excluded. This criterion was applied to ensure that the extracted determinants could be attributed primarily to the e-learning component, rather than reflecting implementation issues related to other concurrent intervention components. Studies addressing topics unrelated to infection management were also excluded.

#### Context

Studies conducted in primary healthcare settings were included. For this review, primary care was defined as clinical settings that provide first-contact and ongoing patient care, including general practice (GP), community-based medical care, and long-term care facilities. Services not directly engaged in patient care, such as pharmacies, were excluded.

#### Type of sources

All study designs were eligible, and both peer-reviewed research and grey literature identified through database searches were considered for inclusion.

In addition to the PCC criteria, studies were limited to publications from 2015 onwards to ensure relevance to contemporary e-learning technologies and practices. Studies published in English or in Scandinavian languages were included. Only studies for which full text was available were included.

### Search strategy

Based on the PCC framework and our research question, we assessed the relevance of keywords using an initial PubMed search. In collaboration with an experienced information specialist, we developed a search strategy structured into three blocks corresponding to the PCC elements. During development, we opted not to restrict the search to studies explicitly indexed as infection management, as preliminary testing showed that such restrictions excluded relevant studies, possibly due to inconsistent indexing. To enhance search sensitivity and avoid missing relevant studies, we therefore applied a deliberately broad search strategy. The complete PubMed search string is presented in Appendix 1.

To ensure comprehensive mapping of the literature across relevant disciplines, the search strategy was adapted for Embase, Cochrane Library, CINAHL, and Scopus. In addition, Google Scholar was searched to identify potentially relevant grey literature. No grey literature sources met the eligibility criteria and were therefore not included in the final review.Reference lists of all included studies were screened to identify additional publications. A full search of all databases was conducted on November 8, 2024, and an updated search was performed on October 24, 2025, to ensure identification of the most recent studies.

### Selection of sources of evidence

All search results were imported into Covidence [25], where duplicate records were automatically and manually removed. Before formal screening, a sample of 25 abstracts was jointly reviewed by two researchers to ensure consistency and reliability in the screening process. Title and abstract screening were then carried out independently by two researchers. Full-text screening was conducted by three researchers working in pairs, with each full text assessed independently by two researchers. Any uncertainties were resolved through discussion, and disagreements were settled by consensus or, when necessary, through consultation with the third researcher.

### Data items

The data extraction form [21] captured descriptive characteristics of each included study, including publication details (title, authors, country, journal), study design, evidence source, and data collection period. Information related to the review question was also charted, including study objectives, population, and sample size, the nature of the e-learning intervention (concept), and the contextual setting in which it was implemented. We extracted all reported determinants of implementation, along with descriptions of how these determinants influenced implementation.

Implementation determinants were extracted only when the original studies explicitly described conditions or factors that were reported to influence implementation, uptake, or use of the e-learning intervention in a positive or negative way.

### Data extraction

Data were extracted using a structured charting form developed for this review. Before full extraction began, two researchers pilot-tested the form on a sample of included studies to ensure consistency and clarity. Following this, the extraction form was refined, and all researchers were subsequently provided with an extraction guide to support uniform data collection. Three researchers (LBJ, JNJ, and SHA) conducted data extraction, and since charting in scoping reviews is an iterative process, the researchers met regularly to discuss uncertainties.

### Synthesis of results

Following data extraction, all determinants related to the implementation of e-learning interventions were coded and organized using the updated Consolidated Framework for Implementation Research (CFIR) [26]. This framework-based approach enabled a structured synthesis, allowing determinants to be mapped across CFIR’s five domains *(Innovation*,* Outer setting*,* Inner setting*,* Individuals*, and *Implementation process*), thereby presenting the findings in a coherent and comprehensive structure (26).

Determinants were coded one at a time and mapped directly to the CFIR domain, subdomain, and construct that best reflected how the determinant was described in the original study. Coding was conducted independently by two researchers (LBJ and CSC). Discrepancies were discussed with a third researcher (SHA) and resolved by consensus.

## Results

### Study selection

**Fig. 1:**
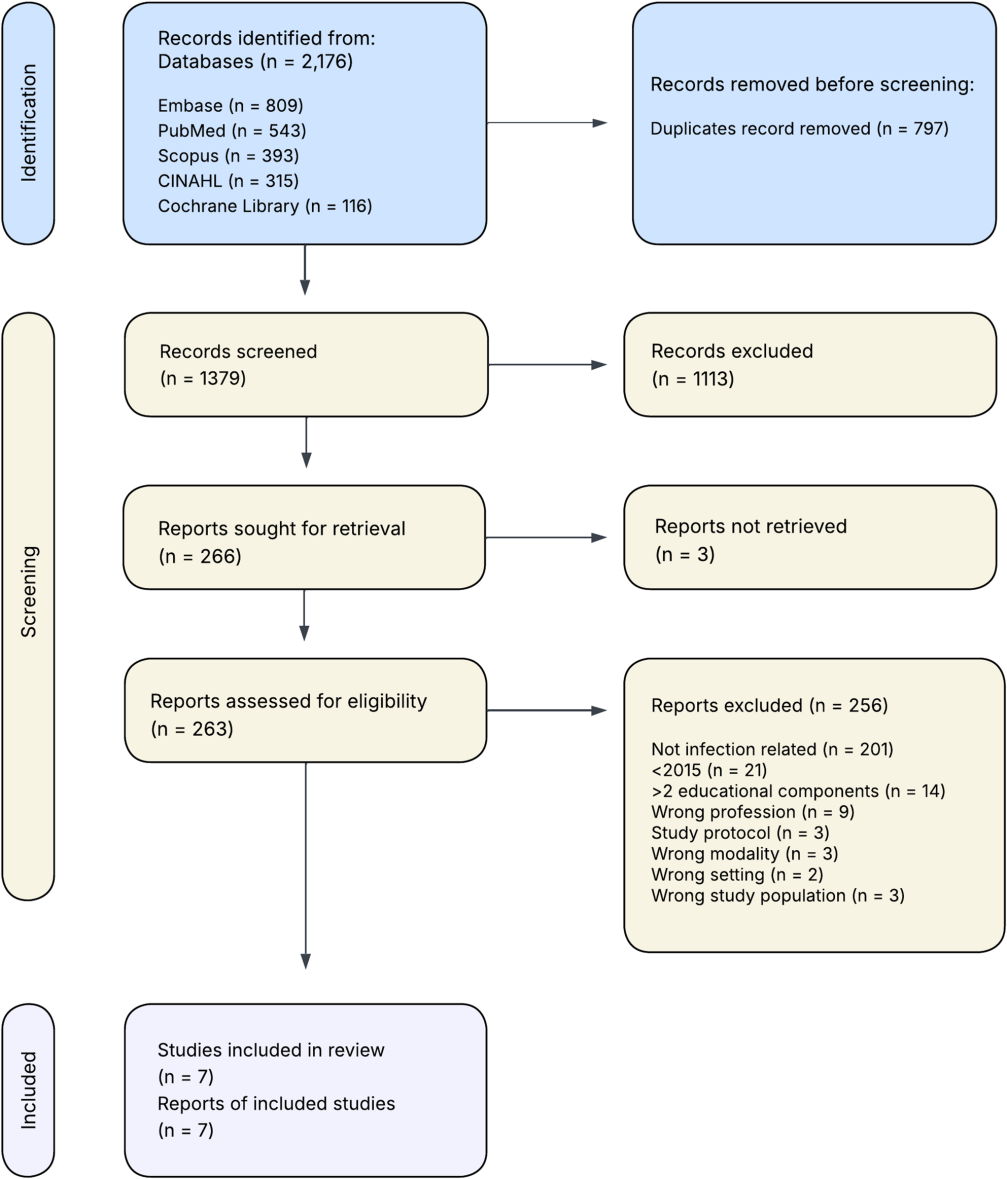
PRISMA diagram of study selection process

A total of 2176 records were initially identified. After removing 797 duplicates, 1379 records were screened based on title and abstract. Of these, 263 full-text articles were assessed for eligibility, and 256 were excluded for not meeting the inclusion criteria. The main reasons for exclusion were publication before 2015, lack of relevance to infection management, inclusion of more than two educational components, wrong setting or target population, wrong study design (e.g., protocols), or incomplete full texts (Fig. 1). The total number of studies included was seven [27–33].

### Characteristics of included studies

Seven studies published between 2017 and 2025 were included in this scoping review. These studies were conducted in the Netherlands [29], Belgium [32], the United Kingdom [28, 33], the United States [30, 31], and Nigeria [27]. Six studies used quantitative designs [27, 29–33], and one used a mixed-methods approach combining online surveys with semi-structured interviews [28]. Data collection was primarily based on online questionnaires, pre- and post-tests, or electronic surveys. Participants across the included studies were HCPs working within primary healthcare and related community-based settings, including GPs and GP trainees [29, 32], nurse- and pharmacist prescribers [28, 33], nurse practitioners [31], and multidisciplinary teams of physicians, nurses, and public-health staff [27, 30]. Characteristics of the seven included studies are presented in Table 1.

**Table 1:** Characteristics of the seven included studies

Study	Country	Study design	Data source	Objective(s)	Population (*n*)	Concept	Context
Bos-Bonnie et al. (2017) [29]	The Netherlands	Quantitative (prospective cohort, pre-post)	Questionnaires	To evaluate if the individual and online e-learning program “The STI-consultation”, using the Commitment-to-Change method, is able to improve knowledge, attitude, and behavior of Dutch General Practitioners, concerning STI-consultations.	GPs and GP-trainees (*n* = 2192 (baseline), 249 (follow-up)	An individual e-learning program “The STI-consultation” using three didactic methods: (1) quick scan with questions on knowledge and attitudes towards STI-consultation, (2) short, interactive videotape case-studies with examples of good and less appropriate behavior, and (3) the CtC-method for follow-up.	General practice
Hale & Lidofsky (2023) [30]	United States	Quantitative (retrospective pre-post analysis)	Questionnaires: pre- and post-curriculum short-term knowledge assessment exam.	To implement and analyze the impact of a hepatitis C virus (HCV) curriculum for primary care professionals across the state of Vermont, USA	Physicians (*n* = 15), nurse practitioners (*n* = 8), nurses (*n* = 8)	A free, open-access, online curriculum pertaining to HCV care, specifically in Vermont. The curriculum consisted of four online modules: (1) HCV basics, (2) screening for HCV, (3) care for patients who screen positive for HCV, and (4) linkage to care and HCV treatment basics. Also, didactic sessions on HCV, both in-person and remotely.	Primary care
Hawker et al. (2025) [33]	United Kingdom	Quantitative (pre-post with follow-up, feasibility study)	Questionnaires	The aim was to assess intervention feasibility and the perceived impact on the prescribing behavior of nurses, pharmacists, and allied health professionals for URTIs in primary care	Nurses (*n* = 18), pharmacists (1), and non-medical other health professionals (*n* = 2) in primary care	The theory-based intervention comprised a 5-minute, interactive, animated scenario of a consultation by a nurse prescriber with a female adult presenting with a URTI. The prescriber depicted in the animation adopted a patient-centered motivational interviewing style to reach a no antibiotic prescribing outcome. To facilitate active learning, non-medical prescribers (NMPs) were invited to test their knowledge at the end of the scenario by answering a range of closed questions that focused on the information presented in the scenario.	Primary care
Lim et al. (2020) [28]	United Kingdom	Mixed method (pre-post experimental + qualitive interviews)	Online survey and semi-structured interviews	To assess the acceptability and feasibility of using a theory-based electronic learning intervention designed to support appropriate antibiotic prescribing by nurse and pharmacist independent prescribers for patients presenting with common, acute, uncomplicated self-limiting respiratory tract infections (RTIs).	Nurses (*n* = 11) and pharmacists, non-medical independent prescribers managing patients with RTIs (*n* = 4)	A theory-based, brief interactive animation electronic learning activity comprised a consultation scenario by a prescriber with an adult presenting with a common, acute, uncomplicated self-limiting RTI to support a *‘no antibiotic prescribing strategy’.*	Primary care
Shane, M. (2021) [31]	United States	Quantitative (quasi-experimental pre-post)	A 10-question electronic pre-test and demographic collection form using Qualtrics Internet-based survey platform	To evaluate the knowledge of NPs working in LTC settings prior to and after using an eLearning module (Antimicrobial Stewardship in Long-Term Care Facilities: Focus on Suspected UTI	Nurse practitioners (*n* = 10)	An e-learning module geared towards NPs in LTCFs.	Long-term care facilities
Thomas et al. (2022) [27]	Nigeria	Quantitative (pre-post, single group)	Survey data and analysis from the platform	To evaluate the first pilot of a modular, self-paced, mobile-ready, and work-relevant online course covering foundational infection prevention and control concepts	Healthcare workers, including doctors, nurses, administrators, Ministry of Health officials, and non-governmental organization employees (*n* = 372 enrolled, 220 completed)	A prototype comprising 10 modules; each requires 10 to 20 min of work. All modules and supporting materials were in English. The first and last modules were the pre-test and post-test respectively; these tests had no time limit and contained the same questions.	Primary healthcare facilities in Nigeria that are typically not staffed by physicians
Van Oost et al. (2024) [32]	Belgium	Quantitative (controlled pre-post study)	Online survey	To assess the self-assessed and general knowledge of GPs regarding PrEP care and evaluate the impact of an e-learning module on GPs’ knowledge about PrEP	GPs (*n* = 58) and GP-trainees (*n* = 14)	Knowledge enhancement about HIV prevention and pre-exposure prophylaxis (PrEP) through e-learning	General practice

The e-learning interventions varied in duration, interactivity, and theoretical underpinning but shared an overall focus on infection management. Most studies evaluated knowledge acquisition and self-reported confidence or attitudes as primary outcomes [28–33], while some additionally measured behavioral intentions or self-reported changes in their practice [28, 29]. Most included studies were pilot or feasibility evaluations of newly developed e-learning interventions [27, 28, 31, 33]. Two studies reported early-stage implementation and exploratory assessment of online curricula [30, 32], and one was a large-scale evaluation of an established national e-learning programme [29].

Although not all included studies explicitly examined implementation processes, all contained information related to factors influencing implementation. While some studies reported such determinants directly as barriers or facilitators [27, 28, 33], others described the feasibility and contextual aspects that shaped the uptake of e-learning interventions [29–32]. Collectively, the findings capture a range of determinants affecting the implementation of e-learning for infection management in primary healthcare.

### Results structured by CFIR domains

The results are presented according to the CFIR domains and organized into facilitators and barriers.

### Innovation

Determinants mapped to the Innovation domain described characteristics of the intervention that influenced implementation. Across the included studies, reported factors spanned *adaptability*, *complexity*, *cost*, *design*, and *relative advantage*.

#### Facilitators

Facilitators within the Innovation domain were mainly related to the *design* of e-learning interventions, where features that simplified access and enhanced usability were reported to promote implementation [27–30]. User-friendly interfaces, mobile-ready platforms, and optimization for low-bandwidth environments improved accessibility [27]. Straightforward access and a simple registration process were also reported to minimize entry barriers [27]. Interactive and scenario-based formats, including realistic video case studies, supported learner engagement and knowledge retention through feedback and contextually relevant examples [27–29]. In one study, the intervention was described as applicable to participants’ professional roles, reflecting alignment between the course content and the participants’ clinical work, which supported its perceived usefulness [28]. Facilitators on the *adaptability* of e-learning interventions included opportunities for self-paced learning, flexible access, and modular course structures that could be completed in short, focused segments [27, 28]. Further, one study reported that a concise, practical course aligned with core infection prevention competencies supported participation and uptake among HCPs [27]. Two studies also highlighted some r*elative advantages* of e-learning over traditional formats, as the flexibility and reach of e-learning were valued in geographically dispersed and resource-limited contexts, where digital delivery provided convenient access to learning opportunities [29, 30]. The online format also enabled timely updates to course content, thereby maintaining alignment with current clinical guidelines [30]. Finally, one study noted that offering the e-learning programme at no *cost* supported participation by removing financial barriers [27].

#### Barriers

Barriers to the implementation of e-learning interventions within the Innovation domain related to issues with *adaptability*,* complexity*,* design*, and *relative advantage.* Several studies identified limited *adaptability* as an implementation barrier, as course content was not always aligned with participants’ professional roles or level of expertise [27, 28]. E-learning modules that mostly focused on practices relevant to tertiary care settings or included basic, non-clinical material were perceived as less useful in routine clinical contexts [27]. In some cases, the educational level was considered too elementary for experienced practitioners, which reduced its perceived relevance [27, 28]. The self-directed and independent study format characteristic of e-learning was described as challenging, offering limited opportunity for interaction guidance during the learning process [31].

Technical and structural aspects of the e-learning platforms also influenced the implementation. In one study, users described systems with multiple menus and screens that were difficult to navigate, indicating that excessive platform *complexity* reduced usability and hindered engagement [27]. Access was further constrained by lengthy registration procedures and requirements to download additional software [27]. The nature of e-learning formats also introduced design challenges, as a digital format inherently offers limited opportunities for social interaction [29]. One study also noted that online courses often relied mainly on passive learning formats, primarily video and text, with few opportunities for active engagement or interaction, leaving few opportunities for active participation or feedback [27]. In one study, difficulties in re-engaging with the intervention over time were reported, as participants were unable to access the e-learning content after completing the intervention [33]. Finally, one study found that even with the inclusion of updated clinical information, the perceived *relative advantage* of the e-learning intervention remained limited because the content was not regarded as new [33].

### Outer setting

Two determinants were mapped to the Outer setting domain, both representing external barriers to the implementation of e-learning interventions. One barrier related to *local conditions* influencing access to the e-learning intervention where firewall restrictions limited access to the programme from clinical settings [28]. Another barrier also reflected *local conditions* in the external environment, as reliance on mobile devices and mobile broadband in a Nigerian primary healthcare context was described as a challenge for implementation. Limited or variable network coverage, together with constraints such as data costs and device access, were reported to hinder consistent engagement with the e-learning intervention [27]. No studies identified facilitators related to this domain.

### Inner setting

Determinants within the Inner setting domain related to organizational conditions relevant to the implementation of e-learning interventions in primary care settings.

#### Facilitators

Facilitators within the Inner setting domain were identified in two studies and related to the compatibility of e-learning within existing work routines [28, 29]. The e-learning modules were considered practical in length and structure, as they were short and organized into modules that could be completed independently, allowing participation to be fitted into normal working schedules or undertaken in brief sessions during the workday [28]. A flexible format also enabled participation alongside other professional responsibilities, as the e-learning intervention could be completed in brief sessions at the participants’ own pace, supporting integration into the workplace context [29].

#### Barriers

Barriers of the Inner setting were related to the organizational context in which e-learning interventions were implemented. When e-learning was combined with live webinars, limited *compatibility* with existing work routines became apparent, as scheduling conflicts with work responsibilities are challenging for participation [27].

### Individuals

Determinants mapped to the Individuals domain described individual characteristics relevant to engaging and applying e-learning interventions. Reported factors primarily related to perceived relevance and individual capability to engage with and apply knowledge and skills through the e-learning format.

#### Facilitators

All identified facilitators within the Individuals domain fell within the subdomain *Characteristics*, reflecting individual *needs*, *capabilities*, and *opportunities* that supported implementation of e-learning interventions. The interventions were perceived as useful for refreshing knowledge, encouraging self-reflection, and supporting changes in professional practice, aligning with participants’ perceived learning *needs* and the perceived relevance of the content for their clinical work [28]. Improved knowledge and skills gained through the e-learning format were reported to enhance individuals’ *capability* to apply relevant concepts in their work, including the use of antimicrobial stewardship strategies to improve clinical practice [31, 32]. Moreover, the *opportunity* to complete training individually and at one’s own pace provided a flexible and comfortable learning environment that encouraged participation, also when addressing sensitive topics, such as consultations revolving sexual transmitted infections [29].

#### Barriers

As with the facilitators, all identified barriers within the Individuals domain were mapped to the subdomain *Characteristics*, specifically within the *capability* construct. In one study, participants who were described as an experienced group of professionals did not perceive e-learning as providing new knowledge or skills [28]. In another study, confusion and frustration when encountering a new or unfamiliar technique were reported as barriers to participation [29].

### Implementation process

Determinants relevant to the Implementation process domain described features related to the engagement and delivery of e-learning interventions.

#### Facilitators

Facilitators within the Implementation Process domain reflected both effective implementation strategies and active participant engagement. In one study, the Commitment-to-Change method was used within an online CME course and described as a helpful strategy to support *implementation*. The method supported the translation of learning into daily practice by prompting participants to formulate personal plans for behavioral change and by providing follow-up reminders several weeks after course completion to encourage reflection on these intentions [29]. In one study, full participation in the intervention was reported, which was interpreted by the study authors as evidence of successful *engaging of innovation recipients* and acceptability of the intervention within the implementation process [33].

#### Barriers

Within the Implementation Process domain, one study identified a barrier related to the distribution format of the e-learning intervention. Although the use of an open online platform in this study was intended to facilitate easier dissemination of the e-learning intervention, it reduced consistent participation [31]. Thus, participants could view only parts of the e-learning material or proceed directly to the post-test, which limited *engaging of innovation recipients*, i.e., reducing the extent to which users interacted with the full intervention [31].

## Discussion

Using the updated Consolidated Framework for Implementation Research (CFIR), this scoping review mapped reported determinants influencing the implementation of e-learning for infection management in primary healthcare settings. Determinants were primarily identified in CFIR’s Innovation and Individuals domains, reflecting a strong emphasis on intervention design and user characteristics. In contrast, determinants related to *Inner settings*, *Outer settings*, and *Implementation processes* were rarely reported. This pattern may be driven by the design and reporting of the included studies, rather than by the true relative importance of these domains. The reported determinants reflect what is described in the included studies and should not be interpreted as indicating which domains are more important.

Across studies, facilitators included design features that supported accessibility and ease of use (e.g., straightforward access, mobile-ready platforms, brief interactive scenarios) [27–30], adaptability (e.g., self-paced modular formats) [27–29], and perceived usefulness for professional roles [28–30]. Barriers included misalignment of content with participants’ expertise [28, 33], platform complexity [27, 31], and limited opportunities for interaction with co-workers inherent to some digital formats [28, 29]. Difficulties in maintaining engagement over time were also noted [31, 33]. A small number of determinants were related to local IT conditions and infrastructure, including firewall restrictions [28] and internet connection issues in resource-limited contexts [27].

Although one study applied an implementation strategy (the Commitment-to-Change method) to support translation of learning into practice [29], and two studies used behavioral design frameworks (COM-B and the Behavior Change Wheel) to inform intervention development [28, 33] none of the included studies used a dedicated implementation framework to plan or evaluate implementation. This suggests that structured implementation planning of e-learning interventions based on insights from implementation science is rare in primary care research on infection management.

In the included studies, determinants related to organizational context, structural conditions, or implementation strategies were less frequently observed than determinants concerning the characteristics of the interventions and their recipients. This pattern aligns with findings from other reviews of e-learning interventions across healthcare settings, in which contextual factors and implementation processes often receive limited attention [17, 34, 35]. This lack of focus contrasts with the broader implementation science literature, which has long established that organizational context, leadership engagement, and structured implementation processes are essential to integration and sustainability of interventions [36, 37]. Only one of the seven included studies employed a qualitative component when assessing the intervention [28]. The remaining studies relied primarily on quantitative designs focused on knowledge retention, knowledge acquisition, or related outcome measures. Consequently, little evidence was available regarding how users experienced interventions, how contextual factors influenced implementation, or how implementation unfolded within primary care organizations.

The limited use of qualitative or process-oriented approaches stands in contrast to the broader developments in implementation science over the past decades, in which qualitative methodologies have increasingly been used to investigate how interventions interact with organizational routines, local conditions, and professional roles [38]. Overall, the review found that the most reported implementation determinants were related to the characteristics of the intervention and its users. Such determinants are also dominant in the wider implementation research literature [26, 39]. Thus, determinants related to the design of the e-learning interventions, particularly those enabling flexibility, easy access, and adaptability to different professional roles, appear to be important in supporting uptake and sustained use. These design-related determinants, however, seem to be conditioned by the availability and functionality of local digital infrastructure, which may either enable or hinder practical implementation.

Most studies indicated that the limited adaptability of e-learning content reduced its perceived relevance, particularly when the materials were either too basic for experienced practitioners or focused on practices from tertiary care settings rather than routine clinical contexts [27, 28]. Such misalignment was reported as a barrier to implementation, suggesting that interventions developed without a clear understanding of participants’ existing competencies and professional roles may be less effective in supporting uptake. While features such as modular structures and self-paced formats were described as facilitators [27–29], these design elements cannot fully compensate for content that does not reflect the learners’ clinical context or level of expertise. Taken together, these findings point to the importance of systematically assessing the target audience during intervention development, including baseline knowledge and professional roles, to enhance the relevance and perceived usefulness of e-learning interventions.

While many identified determinants reflect patterns observed for educational and other complex interventions in primary care more generally [17] e-learning seems to introduce additional determinants that are tied to its digital format and mode of delivery since reliance on IT infrastructure and digital access distinguish e-learning from many other learning interventions. This naturally underscores the need to explicitly consider information technological conditions and prerequisites when planning and implementing e-learning in primary care settings.

### Limitations

The number of studies included was limited, which restricts the extent to which the findings can be generalized across primary care settings. Further, the included studies varied in study design, intervention characteristics, and contexts, including differences in healthcare systems, setting (e.g., general practice and long-term care), and resource conditions, making it challenging to compare across findings. Most of the included studies were not primarily designed to study implementation but focused on investigating the effectiveness of interventions on educational outcomes. Hence, the studies provided limited information on implementation processes, resulting in less insight into organizational and process-oriented determinants. In adherence to the scoping review format, no formal appraisal of methodological quality was conducted, and the scoping review maps reported determinants of implementation of e-learning interventions rather than evaluating the strength of evidence.

Finally, the review focused on published literature indexed in databases considered most relevant for primary care research. While this supported a systematic and transparent search, it may have excluded relevant knowledge reported outside these sources.

## Conclusion

This scoping review identified a limited and heterogeneous number of studies addressing barriers and facilitators to the implementation of e-learning interventions for infection management in primary care settings. Reported determinants were primarily related to intervention design and individual user characteristics, while determinants linked to organizational context, implementation processes, and external conditions were less frequently examined.

Future research should address these gaps by conducting more robust implementation studies that move beyond evaluations of knowledge outcomes and intervention acceptability. There is a need for studies that systematically investigate how e-learning interventions are implemented in primary care settings, including the influence of organizational structures, workflows, and local digital infrastructure.

Qualitative and mixed-methods approaches may be especially valuable for capturing users’ experiences, contextual influences, and implementation processes that are not easily identified through quantitative designs alone. Larger and more diverse studies are also needed to improve the transferability of findings and to support the development of e-learning interventions that are both sustainable and scalable within primary care.

## Data Availability

The dataset supporting the conclusions of this article is included within the article (and its additional files).

## Appendices

### Appendix 1

**Table:** Full PubMed search string

	Participant	Concept	Context
*Text words*	“general practitioner*” “nurse*” “nursing staff” “infection control practitioner*” “nursing assistant*” “health personnel” “healthcare worker” “health professional*” “healthcare professional*” “healthcare assistant*” “healthcare helper*” “nursing home staff”	“e-learning” “elearning” “online learning” “online education” “digital education” “digital learning” “web-based learning” “web-based education” “remote learning” “remote education” “distance learning” “distance education” “hybrid learning” “hybrid education” “blended learning” “electronic learning” “electronic education” “virtual learning” “virtual education”	“nursing home*” “homes for the aged” “old age home*” “primary care” “primary health care” “residential care” “residential facilities”
*MeSH terms*	“General Practitioners” “Nurses” “Nursing Staff” “Infection Control Practitioners” “Nursing Assistants” “Health Personnel”	“Education, Distance”	“Primary Health Care” “Nursing Homes” “Homes for the Aged” “Residential Facilities”

### Appendix 2

**Table:** Mapping of implementation determinants to CFIR domains and constructs

Determinant	Type	CFIR domain/subdomain	CFIR construct
Self-paced - can be accessed at any time [27]	Facilitator	Innovation	Adaptability
Brief - can be broken into short modules [27]	Facilitator	Innovation	Adaptability
Focused and applicable – learning is driven by brief “vignettes” set in healthcare facilities, content focuses on the most fundamental concepts in IPC (infection prevention care), and certificates emphasize job-related course content [27]	Facilitator	Innovation	Adaptability
E-learning was flexible in when and where to perform it [28]	Facilitator	Innovation	Adaptability
Is offered free of charge [27]	Facilitator	Innovation	Cost
User-friendly and simple - has a single-screen and single-button navigation experience that does not require any orientation for learners to get started and is focused on learners’ priorities and experience [27]	Facilitator	Innovation	Design
Mobile-ready and low bandwidth - optimised for mobile devices and for use over 3G connections [27]	Facilitator	Innovation	Design
Immersive learning - learning begins as quickly as possible and is driven by feedback and brief explanations embedded in formative assessment [27]	Facilitator	Innovation	Design
Videotaped case-studies were an important feature of the e-learning program [29]	Facilitator	Innovation	Design
The intervention was applicable and useful to them as prescribers [28]	Facilitator	Innovation	Design
The scenario-based learning approach was well-received; the scenario was realistic and memorable [28]	Facilitator	Innovation	Design
Next to traditional forms of continued medical education, individual and online forms are effective in improving STI-knowledge and attitudes for healthcare professionals [29]	Facilitator	Innovation	Relative advantage
An internet-based curriculum has distinct advantages in a largely rural state like Vermont since a preponderance of its health professionals are located at considerable distances from centers in which in-person learning can be delivered [30]	Facilitator	Innovation	Relative advantage
The curriculum design permits timely revisions in response to evolving updates in hepatitis C virus management, which could not be possible in a static lecture format [30]	Facilitator	Innovation	Relative advantage
Lack of utility - many courses focus on practices appropriate for tertiary care settings or emphasize non-clinical basic science content [27]	Barrier	Innovation	Adaptability
Most prescribers said that the content in the e-learning intervention was pitched at a lower level of experience [28]	Barrier	Innovation	Adaptability
The independent study style of the eLearning module [31]	Barrier	Innovation	Adaptability
Complex user interfaces - some platforms have many different menus, screens and pages that may have limited utility for learners and can interfere with learning [27]	Barrier	Innovation	Complexity
Lengthy registration processes -some platforms have multi-step registration processes or require download of an app [27]	Barrier	Innovation	Complexity
Lack of social activity between participants [29]	Barrier	Innovation	Design
It may be helpful to understand time constraints experienced by nurse practitioners, which may affect the amount of time available to complete eLearning modules [31]	Barrier	Innovation	Design
Passive learning - online courses often rely heavily on video and text, with limited opportunities for interactivity [27]	Barrier	Innovation	Design
A few prescribers reported experiencing problems assessing the intervention at work due to the NHS firewall [28]	Barrier	Outer setting	Local conditions
Mobile devices and connectivity - most users access internet with mobile devices using cellular data [27]	Barrier	Outer setting	Local conditions
The length of the intervention (5-20 min.) Prescribers agreed that the intervention could be completed during work time because it was a short learning session [28]	Facilitator	Inner setting	Combability
It provides educational opportunities at times that do not conflict with clinical responsibilities [30]	Facilitator	Inner setting	Combability
Scheduling issues - Healthcare workers work may conflict with synchronous events (webinars) [27]	Barrier	Inner setting	Combability
Prescribers generally considered the intervention to be useful because it provided the opportunity to remind themselves on the topic, enabled self-reflection and change practice [28]	Facilitator	Individuals/Characteristics	Need
This eLearning module has shown significant increase of knowledge of nurse practitioners in long-term care settings, thereby empowering them to utilize this information and antimicrobial stewardship strategies to improve the safety and quality of older adults in long-term care facility residents [31]	Facilitator	Individuals/Characteristics	Capability
An e-learning modules serves as an effective method to enhance the knowledge and skills of GP in the realm of PrEP care [32]	Facilitator	Individuals/Characteristics	Capability
The majority of prescribers did not think that they had acquired new knowledge or skills from the e-learning intervention because they were largely an experienced group of practitioners [28]	Barrier	Individuals/Characteristics	Capability
Confusion or frustration as a result of new and unknown technique [29]	Barrier	Individuals/Characteristics	Capability
The Commitment-to-Change method might be effective in individual online CME-courses [29]	Facilitator	Implementation Process	Reflecting and evaluating
The use of YouTube as the platform for distribution. The slideshow was uploaded to YouTube with the intention of facilitating easier dissemination to participants; however, participants may choose to watch only part of the eLearning module, and they have the ability to access the posttest without first completing the entire eLearning module [31]	Barrier	Implementation process	Engaging
